# Supplementary material for: Study design and baseline to evaluate water service provision among peri-urban communities in Kasai Oriental, Democratic Republic of the Congo
Source: PLoS One. 2023 Apr 13;18(4):e0283019. doi: 10.1371/journal.pone.0283019 (PMC10101432; doi:10.1371/journal.pone.0283019)
Supplement: S1 File — (PDF) [file pone.0283019.s001.pdf]

Office of Research Integrity
UNIVERSITY OF COLORADO **BOULDER**
INSTITUTIONAL REVIEW BOARD

Institutional Review Board
563 UCB
Boulder, CO 80309
Phone: 303.735.3702
Fax: 303.735.5185
FWA: 00003492

APPROVAL

19-Apr-2021

Dear Evan Thomas,

On 19-Apr-2021 the IRB reviewed the following protocol:

Type of Submission:	Amendment
Review Category:	Expedited
Risk Level:	Minimal
Title:	Impact Evaluation of Water and Sanitation Providers in Democratic Republic of Congo
Investigator:	Thomas, Evan
Protocol #:	20-0491
Funding:	Federal
Documents Approved:	20-0491 Consent Form - Eng (19Apr21); Household DRC Survey_English.pdf; 20-0491 Protocol (19Apr21); Household DRC Survey_French.pdf; 20-0491 Consent Form - French (19Apr21);
Documents Reviewed:	IRB Comments tracking.xlsx; HRP-213 Amendment-v3;
Description:	- Submission of full Study Design, procedures and supporting documents for approval per determination under 45CFR46.118. Approved per Expedited review categories 4 and 7.
Notes:	- No human research may be conducted under this study prior to submission of the Lomami and Kasai Oriental Province Provincial Health Division for Democratic Republic of Congo study approval.<br><br>- Non-CU Boulder personnel are not to be involved in human research under this study prior to the submission of an IRB Authorization Agreement with the investigator's home institution.

IRB Approval for this protocol will expire on **18-Apr-2022**.

You are required to use the IRB Approved versions of study documents to conduct your research. The IRB Approved documents can be found here: Approved Documents

In conducting this protocol you must follow the requirements listed in the INVESTIGATOR MANUAL (HRP-103).

Sincerely,
Douglas Grafel
IRB Admin Review Coordinator
Institutional Review Board

This letter has been electronically signed in accordance with all applicable regulations, and a copy is retained within the University of Colorado Boulder's IRB records.
