# Supplementary material for: Study design and baseline to evaluate water service provision among peri-urban communities in Kasai Oriental, Democratic Republic of the Congo
Source: PLoS One. 2023 Apr 13;18(4):e0283019. doi: 10.1371/journal.pone.0283019 (PMC10101432; doi:10.1371/journal.pone.0283019)
Supplement: Attachment — Submitted filename: PLOS One Submission Review.pdf [file pone.0283019.s002.pdf]

PLOS One Submission Review

Journal Requirements:

When submitting your revision, we need you to address these additional requirements.

1. Please ensure that your manuscript meets PLOS ONE's style requirements, including those for file naming. The PLOS ONE style templates can be found at https://journals.plos.org/plosone/s/file?id=wjVg/PLOSOne_formatting_sample_main_body.pdf and https://journals.plos.org/plosone/s/file?id=ba62/PLOSOne_formatting_sample_title_authors_affiliations.pdf

We used the Plos One latex template and have confirmed style requirements.

2. Please include a complete copy of PLOS' questionnaire on inclusivity in global research in your revised manuscript. Our policy for research in this area aims to improve transparency in the reporting of research performed outside of researchers' own country or community. The policy applies to researchers who have travelled to a different country to conduct research, research with Indigenous populations or their lands, and research on cultural artefacts. The questionnaire can also be requested at the journal's discretion for any other submissions, even if these conditions are not met. Please find more information on the policy and a link to download a blank copy of the questionnaire here: https://journals.plos.org/plosone/s/best-practices-in-research-reporting. Please upload a completed version of your questionnaire as Supporting Information when you resubmit your manuscript.

Enclosed.

3. Please amend your current ethics statement to address the following concerns:

a) Did participants provide their written or verbal informed consent to participate in this study?

Updated. "...As approved by the review boards, survey participants provide verbal informed consent during each survey and confirm they are at least 18 years of age and are knowledgeable about household water practices. Consent is marked in the mWater surveyor in enumerator tablets."

b) If consent was verbal, please explain i) why written consent was not obtained, ii) how you documented participant consent, and iii) whether the ethics committees/IRB approved this consent procedure.

ii and iii completed: "...As approved by the review boards, survey participants provide verbal informed consent during each survey and confirm they are at least 18 years of age and are knowledgeable about household water practices. Consent is marked in the mWater surveyor in enumerator tablets."

Verbal consent was chosen and approved by the IRB both because of limited literacy and the low-risk of participation.

Please review your reference list to ensure that it is complete and correct. If you have cited papers that have been retracted, please include the rationale for doing so in the manuscript text, or remove these references and replace them with relevant current references. Any changes to the reference list should be mentioned in the rebuttal letter that accompanies your revised manuscript. If you need to cite a retracted article, indicate the article's retracted status in the References list and also include a citation and full reference for the retraction notice.

All references confirmed.

Reviewers' comments:

Reviewer #1: This paper is not recommended for publication for the following reasons:

1. It presents a study design rather than a completed study. The novel contributions to knowledge are limited, since most of the presented study design draws on established methodologies, with few new methods.

This paper is submitted as a study design consistent with the guidelines provided by PLOS One for study design papers.

2. There is no information about the budget available to conduct this study nor the personnel available. It is difficult to judge therefore how applicable this study design would be to other settings.

These resource considerations are not typically included in study design papers that we are aware of.

3. There is no information about the ethical considerations inherent in the study and how these will be addressed. For example, if the health assessment identifies poor health in the study subjects, will they be offered treatment? Same for other aspects of the study (e.g. water quality).

This is not directly relevant for a study of an active water and sanitation implementation. This is not a clinical trial.

4. The paper should have discussed the possible downsides/biases of their approaches and which alternative approaches were considered and dismissed. That would have been a more useful contribution to the literature on this subject.

The abstract and introduction do discuss briefly challenges with RCTs ("Randomized controlled trials, for example, can also be prohibitively expensive for programs and require additional rigidity during implementation. There is a need for implementation science which can achieve causal inference, while allowing the required targeted interventions flexibility during implementation.")

Reviewer #2: This manuscript describes a well-designed protocol for evaluating the impact of water service provision inteventions in DRC using an implementation science framework. The proposed study design and analytical methods are sound and will be an important contribution to the evidence base for WASH intervention effectiveness.

Major comments

• The manuscript would benefit from a clear and early (e.g. in Background section) description of the research question, including 1) the intervention activities, 2) outcomes being evaluated.

While this is also in the "Study Rationale" section, we have clarified this in the first sentences of the last background paragraph:

"Ultimately, this study seeks to pilot and assess an implementation science impact evaluation approach in the areas immediately surrounding 14 water service providers, utilizing a variety of short-, medium-, and long-term monitoring and impact evaluation methods to provide feedback at multiple points during the intervention. Planned intervention activities will be specific to the needs of each WSP, and may include infrastructure rehabilitation, training in technical maintenance, support for financial management and sustainability, assistance with administration, and bolstering of WSP governance."

• The manuscript presents preliminary data, but this is not mentioned in the title, abstract, or background section. I suggest re-framing this paper as "study design and preliminary baseline results"

We have revised the title to "Study design and baseline to evaluate water service provision among peri-urban communities in Kasai Oriental, Democratic Republic of the Congo"

Minor comments:

• Verb tense changes throughout, particularly in the design section. Since these decisions presumably have already been made (and likely implemented), use of past tense is suggested.

Updated.

• Suggest including line numbers to facilitate next round of review

Updated.

• Make sure all acronyms are defined at first use

Updated.

• The preliminary results often repeat methods earlier described; methods should not be included in results section.

Updated.

Specific comments

Abstract:

• "…to establish the impact of interventions on peri-urban water service provision" – please define specifically your outcomes of interest (water provision is pretty broad)

Updated to, "on peri-urban water access, security and quality"

• Briefly include information on sampling and data collection methods – for example household-based measures are referenced (water security, health status), but how many households will be surveyed, and how will they be selected?

Updated

• Final sentence: What are the program outcomes and how will success be defined?

Updated to, "lead to improved water access, security and quality."

Background

• Define WASH at first use

Updated

• Paragraph 6 (about ITS) would fit better in the methodology section

After further revision of the paper, we believe that this paragraph is appropriate in the background as it contextualizes the approach. The actual study design is presented in the methodology section as suggested.

• The intervention and research question is unclear. Please state what the specific intervention(s) are and what specific outcomes are being evaluated.

Again, we added from above "Planned intervention activities will be specific to the needs of each WSP, and may include infrastructure rehabilitation, training in technical maintenance, support for financial management and sustainability, assistance with administration, and bolstering of WSP governance" but this is an inherent challenge of this design.

Design

Study setting

• How were the 32 WSPs selected?

Corrected to, "Ultimately, 32 WSPs were selected by Chemonics for inclusion in the intervention program based on subjective programmatic criteria including need and implementation feasibility."

Study design

• How were WSPs included in study randomly selected?

o Edited to add: this is then described in the subsequent paragraph. This section is therefore repetitive and should be made more concise. No need to state in the first paragraph that you will randomly select WSPs and in the next paragraph that you did select WSPs. Please complete a through review of the methods for conciseness.

Updated.

• Who is going to be targeted for the household survey, and how are they selected? What happens if they aren't there for a visit? Can someone else be surveyed?

Expanded to say, "Within each WSP, we are collecting data at six-month intervals on a cohort of 100 randomly selected households for the duration of the study period within 500m of the WSP. The households randomly selected at baseline are retained over the course of the study. Using an open cohort design, if a household is unavailable after three visits, another household is randomly selected. This six-month assessment includes all tools such as the point-of-collection survey, the household survey, and water quality assessments. Water quality testing is performed in a randomly selected sub-sample of 25 households per WSP."

Sample size

• How are you defining diarrhea?

Manuscript updated to clarify - "In the last 7 days (since this day last week), did your child have:" and options include fever, diarrhea, 3 or more bowel movements in a single day, water or soft stool, blood in the stool, or toothache."

Study Rationale

• This is exactly what I was hoping to see in the background section. I suggest moving all but the paragraph starting with "Figure 3" to background so the audience knows what the interventions include and what outcomes you are evaluating

Moved the following two paragraphs into the background:

"Our study seeks to assess the efficacy of interventions to improve the delivery and quality of service provision of these 32 WSPs. Interventions are specific to the existing capacity of each WSP, and may include support to improve infrastructure, financial capacity, institutional governance, and other assistance as needed. To assess these interventions, this research utilizes short- and medium-term monitoring and impact evaluation methods in order to provide feedback at multiple points during the intervention. Short-term methods provide rapid feedback to improve the intervention during the implementation cycle, where medium-term evaluation will assess the intervention from a systems-level perspective.

The rapid feedback monitoring will produce periodic evidence-based reports that demonstrate the outcomes and impacts of water and sanitation delivery activities, including continuity of water services, point-of-consumption and point-of-collection microbial water quality, household water security, measures for household health status, ability and willingness to pay for water service provision, and WSP performance monitoring. Medium-term evaluation focuses on the use of qualitative comparative analysis (QCA) whereby the combination of factors that lead to successful or unsuccessful program outcomes can be posited."

Eligibility

• There is no eligibility criteria described here, other than being within 500 meters of a standpipe operated by the WSPs. Is there other eligibility criteria?

We don't have formal eligibility criteria beyond this, and we listed the informed consent requirements for survey participants elsewhere.

• Suggest moving this up to the study design section

Moved.

Study components

• Suggest clarifying that these surveys are conducted among the selected households.

Updated to "Point-of-collection surveys are conducted at the 14 water service providers and point-of-use surveys are conducted at the selected households every six months. The point-of-collection surveys target nearby users currently collecting water from the water service provider. The following sections describe some of the tools built into these surveys."

• Surveys "on 12 water service providers" wasn't previously described – who are you surveying within these providers?

Updated to "Point-of-collection surveys are conducted at the 14 water service providers and point-of-use surveys are conducted at the selected households every six months. The point-of-collection surveys target nearby users currently collecting water from the water service provider. The following sections describe some of the tools built into these surveys."

• Please state whether poverty scorecard tool is validated (+ citation, if available)

Revised.

• WTP evaluation – (last sentence) – will the DCE evaluation be conducted among the selected households or elsewhere? This is unclear based on current phrasing.

Updated to flag selected households.

• Health – how is diarrhea defined

Manuscript updated to clarify - “In the last 7 days (since this day last week), did your child have:" and options include fever, diarrhea, 3 or more bowel movements in a single day, water or soft stool, blood in the stool, or toothache.”

o What do you mean by “complemented by public health records”? Do you mean verify with medical records for illness events?

We specified “community-level” public health data.

• Water quality assessments – 25 households per WSP in the study, correct? Not 25 households total. Please clarify and also include overall total of households undergoing water quality assessments.

Updated to reflect 25 households pert WSP every six months, for a total of 300 households undergoing water quality assessments every six months.

• QCA – in the last paragraph in this section, the sentence starting with “we will then assess QCA metrics…” is very hard to follow.

Statistical methods

• All models will be adjusted for both individual and WSP-level characteristics in order to minimize potential selection bias and confounding resulting from the non-random timing of program delivery – suggest giving examples of these characteristics and how they will be determined.

Revised to say, “These include water source type, water quality and reliability as well as categorized management models.”

• 2nd paragraph says “section ?”

Updated.

Preliminary data

• Suggest including in abstract, background (and title) that this paper will also present preliminary baseline data, since this hasn’t been mentioned until this point

Concur. The abstract and title has been updated.

Were the households included in the baseline ultimately enrolled in the study? Unclear if this is a part of the larger study or just preliminary/exploratory work

I can certainly expand on the open-cohort design, but this is a bit complicated as we altered our methodology. Essentially we included 669 households in the Lot 1 baseline but surveyed 100 households at WSPs beyond that. Of these 669, 100 households per WSP will be randomly selected to continue. Best practices on articulating a methodology change in this paper?

• How many households were included in the baseline survey? Is it 669 (as specified in the demographics and poverty scorecard section?) if so, please move up as it is unclear whether 669 is how many surveys were administered or how many completed the scorecard.

Will be updated in the final count (adding in other WSPs).

Moved to the summary source.

• How is “primary water source” defined?

Participants are asked to indicate their main source of drinking water used by the household at that time. Primary water options include a public tap, protected spring, unprotected dug well, bottled water, rainwater, river, and other common options.

• Please also present water sources as categorized improved/unimproved since that was what was described in the methods.

Updated.

• Willingness to pay – consider conversion to USD or present conversion rate.

Updated.

• MUAC – the first part of this paragraph (defining MUAC and how it’s measured/used) belongs in the methods.

Updated

• Water quality assessments – how many households total?

Updated.

• Water quality assessments – the final paragraph (describing JMP criteria) belongs in methods

Updated.

Discussion

• Last sentence of first paragraph contains typos

Updated.

• The estimation strategies belongs in methods – do not introduce new ideas in discussion section

Moved to the statistical methods section.

Reviewer #3: The manuscript presents the study protocol for an evaluation of a USAID intervention designed to improve water service provision in peri-urban communities in two provinces in the Democratic Republic of the Congo. The study aims to overcome challenges

inherent with some monitoring practices that fail to assess the overall project performance. It pilots an implementation science impact evaluation approach to attempt to overcome the issues associated with traditional impact evaluations (e.g. the lengthy processes). The study is designed to assess performance against multiple indicators and will use a range of data collection methods. The study is timely, as it identifies the tension that exists in monitoring and evaluation (M&E) between prompt feedback that can contribute directly to project activities, and rigour.

The manuscript could be strengthened by developing the opening paragraphs of the Background section. Stronger engagement with previously published research would enhance the section and provide clearer context. It appears that there is a strong rationale for research into how best to balance M&E rigour with prompt results but refining and tightening the Background section would help this message to come to the fore. Relatedly, further information on the intervention that the study is designed to evaluate would be useful for context. While more information on the intervention as a whole would be beneficial, specific details on whether all the water service providers in the area will receive an intervention would be helpful, as would clarity on the timing of the intervention in relation to the data collection. Details of whether any other interventions are taking place or scheduled to take place that could impact the study would also be useful.

We have added the following details in the Background section, "Planned intervention activities will be specific to the needs of each WSP, and may include infrastructure rehabilitation, training in technical maintenance, support for financial management and sustainability, assistance with administration, and bolstering of WSP governance."

On a related point, clarification on whether information will be obtained to identify changes in the water point that participants use, factors driving those changes and whether/how changes in water point could impact the inferences of the study would be beneficial. The wording of the Study components section suggests that the methods are not comprehensively outlined in the protocol. The description needs to provide adequate details for the protocol to be reproduced and replicated. Further information may be required to achieve this. In Figure 3, for example, it looks like remote sensing climate/weather data will be measured but this is not elaborated on in the text.

Concur. In the revised paper, we have clarified throughout the outcomes, measures and analysis performed, and ensured that the manuscript and Figure 4 are aligned, including the statement, "Further, we will incorporate geospatial and temporal characteristics including rainfall to establish any seasonality attributes associated with the outcomes measured."

The manuscript would benefit from further proofreading. For example, the acronym WASH and the initialism ETD should be spelt out in full in the first instance. In Section 4 (statistical methods), on the third line of the second paragraph, authors are signposted to a section, but the specific section is marked by a question mark. In the references list, missing information in reference 34 is marked by question marks and reference 45 looks to be incomplete, with the URL in the main body of the text, but limited information in the references list.

Updated.

On a minor point, Figure 2 could perhaps be reformatted to improve clarity. Deleting the empty rows, for example, may make the schedule look cleaner.

Clarification on the form of consent obtained (written/oral) and when it will be obtained would also strengthen the manuscript. There appears to be limited information on the data management plan, so further details on this would be useful.

Updated in Ethics: “This study was reviewed and approved by the University of Colorado Boulder Institutional Review Board and the Lomami and Kasai Oriental Provincial Health Division. As approved by the review boards, survey participants provide verbal informed consent during each survey and confirm they are at least 18 years of age and are knowledgeable about household water practices. Consent is marked in the mWater surveyor in enumerator tablets and data is managed in mWater.”
