# Supplementary material for: Study design and baseline to evaluate water service provision among peri-urban communities in Kasai Oriental, Democratic Republic of the Congo
Source: PLoS One. 2023 Apr 13;18(4):e0283019. doi: 10.1371/journal.pone.0283019 (PMC10101432; doi:10.1371/journal.pone.0283019)
Supplement: Attachment — Submitted filename: PLOS One Submission Review (1).pdf [file pone.0283019.s003.pdf]

Reviewer #2: A few minor comments:

• First sentence in abstract - recommend adding “results” after “We present study design and baseline”

Updated.

• Lines 345-349 remains very difficult to follow. Suggest breaking into sentences for each component of the QCA metrics.

Updated.

• Line 423 – suggest replacing the word “significant” with another adjective because of connotation with “statistically significant”, particularly since this is the results section

Updated.

• Line 544 – this should be effectiveness not efficacy

Updated.

Reviewer #3: Thank you for taking the time to address many of the comments. The manuscript could be enhanced further by making additional edits to the Introduction. For example, lines 71 - 75 are very similar to lines 88 - 94 so there is some slight repetition there. Addressing this, and restructuring the Introduction section more broadly could improve the flow and focus of the initial part of the manuscript. Also, a further check through the in-text citations to ensure that all relevant information is referenced, would be beneficial. It is unclear, for example, where the information provided in lines 46/47 was obtained from.

Updated.
